# Frequency, clinical presentation and management of primary amenorrhea in a tertiary care setting

**DOI:** 10.12669/pjms.40.11.9317

**Published:** 2024-12

**Authors:** Sumera Khan, Shehla Arif, Farah Deeba Nasrullah, Riffat Jaleel

**Affiliations:** 1 Sumera Khan, FCPS, Department of Obstetrics and Gynaecology Unit II, D. Ruth K. M. Pfau Civil Hospital Karachi and, Dow University of Health Sciences, Karachi, Pakistan; 2 Shehla Arif, FCPS, Department of Obstetrics and Gynaecology Unit II, D. Ruth K. M. Pfau Civil Hospital Karachi and, Dow University of Health Sciences, Karachi, Pakistan; 3 Farah Deeba Nasrullah, FCPS, MRCOG, Department of Obstetrics and Gynaecology Unit II, D. Ruth K. M. Pfau Civil Hospital Karachi and, Dow University of Health Sciences, Karachi, Pakistan; 4 Riffat Jaleel, FCPS, Department of Obstetrics and Gynaecology Unit II, D. Ruth K. M. Pfau Civil Hospital Karachi and, Dow University of Health Sciences, Karachi, Pakistan

**Keywords:** Primary amenorrhea, MRKH syndrome, Chromosomal abnormalities, Outflow tract obstruction

## Abstract

**Objective:**

We aimed to determine frequency, clinical presentation, etiology and management in patients presenting with primary amenorrhea in tertiary care setting.

**Method:**

This was a case series conducted in outpatient Department of Gynecology and Obstetrics unit II, Dr. Ruth K. M. Pfau Civil Hospital Karachi from 1^st^ July 2019 to 30^th^ June 2022. A total of 20,102 patients attended Gynaecology outpatient department in these three years. We included 41 cases with primary amenorrhea. Information collected on a specially designed proforma included history, physical examination, hormonal workup, ultrasound, radiological investigations and karyotyping results. Data was entered and analyzed by SPSS version 26.0.

**Results:**

The frequency of PA was 41(0.2%). The mean age was 17.93± 4.27 years (range13-37). Majority 38(92.6%) were unmarried, educated till secondary 24(58.5%), of normal height 28(68.2%) and normal BMI 30(73%). Main associated complaint was cyclical lower abdominal pain 8(22%). Most common cause found was Mullerian dysgenesis 17(41.46%). Others were chromosomal disorder 10(24.39%), imperforate hymen 6(14.63%), constitutional delay 5(12.19%), transverse vaginal septum 2(4.87%) and congenital adrenal hyperplasia 1(2.43%). The chromosomal analysis revealed 46XX in 31(75.6%), 45XO/45XO Mosaic in 6(14.63%) and 46XY in 4(9.75%) patients. Surgical correction was possible in 14(41.66%) patients, hormone replacement therapy (HRT) was given in 10(22.2%) and rest treated with placebo.

**Conclusion:**

Primary amenorrhea is a significant problem in adolescent girls. We found Mayer Rokitansky- Kuster- Hauser syndrome (MRKH) syndrome as the commonest cause in our series. There is need to promptly identify the patients who need medical, surgical or psychological management. It is also required to make local strategies and guidelines for evaluation, management and long term follow up.

## INTRODUCTION

Primary amenorrhea (PA) has major social and psychological implications affecting quality of life. Any delay in menstruation generates anxiety in patients and parents. Most frequent concerns are future sexual activity and fertility. An effective psychological intervention or behavioral therapy often needs to be supplemented with clinical treatment. PA is defined as absence of menstruation by 13 years in presence of secondary sexual characteristics (SSC) or by 15 years regardless of development of SSC.^1^ The menstruation is under control of hypothalamic pituitary ovarian (HPO) axis, any disruption in HPO axis or outflow track may lead to absence of menstruation.^2^,^3^

The incidence of primary amenorrhea is less than 1% worldwide with hypothalamic causes accounting for one third cases.^4^ The etiological factors include endocrinological, chromosomal aberrations, outflow tract obstruction and constitutional delay^5^,^6^ SSC are important while making a diagnosis i.e. thelarche, pubarche, adrenarche and menarche.^7^,^8^ MRKHS is a normal female with 46 XX karyotype and absent/rudimentary uterus and it affect about 1:5,000 newborn. The chromosomal aberrations related to sex chromosome may be numerical or structural. Some cases are as a result of mosaicism of X chromosome i.e. X/ XXX and X/ XX.^9^

PA is one of the emerging health problems in adolescents. We analyzed cases of primary amenorrhea aiming to find out the definitive etiology and diverse clinical presentation as differential diagnosis of primary amenorrhea has always been a challenging exercise taking geno and phenotypic development into account. This study will help develop an individualized approach in investigating and treating PA and directing specific interventions based on etiology. Limited national studies are available to know the burden of the disease and our study will contribute valuable information to understand the clinical presentation and differential diagnosis of the problem in our local setup.

## METHODS

It was a case series and the data was collected retrospectively from outpatient department of Gynecology and Obstetrics unit II, Dr. Ruth K. M. Pfau Civil Hospital Karachi .

The information obtained from patients is entered over a proforma routinely in our outpatient. Case records of patients over 13 years with or without the development of SSC were included from 1^st^ July 2019 till 30th June 2022. The patients with secondary amenorrhea and pregnancy were excluded. All data was collected retrospectively by trained postgraduates of our unit.

### Ethical Approval:

The study was approved from institutional review board (IRB-2594/DUHS/2022/1034, dated: September 20, 2022).

Our initial workup was based on comprehensive patient’s history and physical examination, taking into account, presence or absence of SSC, cyclical abdominal pain, urinary or bowel disturbances, marital status, sexual history and previous medical/ surgical/ drug history. Examination included assessment of height, weight, BMI, general physical examination, axillary/ pubic hair and breast development according to Tanner’s staging. Abdominal and external genitalia examination done to determine the labial development, exclude undescended gonads and demonstrate hymenal patency to rule out outflow tract obstruction.

In case of normal SSC, pelvic ultrasound was performed to assess anatomy. If uterus was rudimentary or absent, karyotyping was advised to rule out chromosomal abnormalities. The patients were labelled as MRKHS if it was 46XX and the diagnosis was XY female if 46XY. Presence of uterus necessitated further assessment of outflow obstruction (hematometra/ hematocolpos). Serum FSH, LH and Prolactin were also checked where ultrasound showed normal pelvic anatomy. Normal levels indicated constitutional delay and elevated showed resistant ovary syndrome. Elevated prolactin pointed to prolactinoma, further confirmed by CT scan.

In case of absent or underdeveloped SSC, serum FSH and LH were carried out and karyotyping was requested if their levels found high. High gonadotrophins with 46XX karyotype, indicated premature ovarian failure, resistant ovary syndrome or gonadal agenesis. If karyotype was 46XY, the condition was either 46XY gonadal agenesis or testicular enzymatic failure. Turner’s syndrome or mosaic was diagnosed with 45XO and short height. In patients with low gonadotrophin levels with normal height the diagnosis of hypo-gonadotrophic hypogonadism was made while those with short height, suspicion of intracranial lesion was raised.

In patients where sign and symptoms suggestive of hyperandrogenism present, serum free testosterone was done. In selected cases 17 OH-progesterone levels were also investigated. MRI was only carried out to determine associated abnormalities in mullerian agenesis or to locate gonads in cases of XY female. The patients were investigated as per recommendation of Dewhurst’s Textbook of Obstetrics and Gynaecology.^10^ Data was analyzed on SPSS 26. Frequencies and percentages were calculated for qualitative variables while mean and standard deviation for quantitative variables.

## RESULTS

A total of 20,102 patients attended gynecological outpatient department during three years of our study and 41(0.2%) presented with primary amenorrhea. All were phenotypically females.

### Demographic characteristics:

The mean age was 17.93± 4.27years (range 13-37 years). Majority 38(92.7%) of them were unmarried and were urban residents 36(87.8%). About 5(12.1%) were educated till primary, 24(58.5%) till secondary and rest were uneducated. All of our patients with 46XX karyotype and majority of MRKH patients (76%) had normal height. A large number of (83.3%) patients with Turner were short and all XY females were tall. Most of them 30(73.1%) had BMI within normal range, 7(17%) were underweight and 4(9.7%) were overweight.

### Presenting features:

In addition to failure to menstruate other associated complaints were cyclical abdominal pain 8(19.5%), subfertility and apareunia 3(7.3%) and other presentations like ambiguous genitalia, inguinal swelling and voice changes were 5(12.1%).

### Etiology:

The causes detected were MRKHS 17(41.46%), chromosomal disorder 10(24.39%), imperforate hymen 6(14.63%), constitutional delay 5(12.19%), transverse vaginal septum 2(4.87%) and congenital adrenal hyperplasia 1(2.43%). The chromosomal analysis revealed 46XX in 31(75.6%), 45XO/45XO Mosaic in 6(14.63%) and 46XY in 4(9.75%) patients. SSC of the participants are given in Table-I. Clinical and sonological characteristics of genital tract are given in Table-II. Various modes of provided management are listed in Table-III. Counselling, reassurance, psychological support and nutritional supplements given in rest of the cases.

**Table-I:** Distribution of secondary sexual characteristics in different etiologies.

Causes	Frequency n	Axillary hair n(%)	Pubic hair n(%)	Breast^1^ n(%)
Mullerian Agenesis	17	10(58.3)	10(58.3)	
Developed				10(58.3)
Less developed		7 (41.17)	7(41.17)	7(41.17)
Outflow Tract Obstruction	8	8(100)	8(100)	
Developed				6(75)
Less developed		0	0	2(25)
Turner/Turner Mosaics	6	1(16.67)	1(16.67)	
Developed				1(16.67)
Less developed		5(83.33)	5(83.33)	5(83.33)
Constitutional delay	5	5(100)	5(100)	
Developed				5(100)
Less developed		0	0	0
Xy-gonadal dysgenesis	4	1(25)	1(25)	0
Developed				
Less developed		3(75)	3(75)	4(100)
Cah	1	0	0	0
Developed				
Less developed		1(100)	1(100)	1(100)

Developed: Tanner ≥ 3; Less developed: Tanner<3.

**Table-II:** Clinical and sonological assessment of genital tract.

CAUSES	Frequency n	Ovaries n (%)	Uterus n (%)	Vagina n(%)	Genitalia^3^ n (%)
MRKHS					
Normal	17	10(58.8)	1(5.88)	12(70.58%)^1^	15(88.24)
Streak/Rudimentary		5(29.41)	13(76.47)	5(29.41%)^2^	2(11.76)
Absent		2(11.76)	3(17.64)	-	-
Outflow obstruction	8				
Normal		8(100)	8(100)	6(75)	8(100)
Streak/Rudimentary		0	0	2(25)^2^	0
Absent		0	0	-	-
Turner/turner mosaics	6				
Normal		0	0	6(100)	5(83.33)
Streak/Rudimentary		4(66.67)	5(83.33)	0	1(16.67)
Absent		2(33.33)	1(16.67)	-	-
Constitutional delay	5				
Normal		5(100)	5(100)	5(100)	5(100)
Streak/Rudimentary		0	0	0	0
Absent		0	0	-	-
XY-gonadal dysgenesis	4				
Normal		0	0	2(50)	3(75)
Rudimentary/ Blind End^3^		2(50)	1(25)	2(50)	1(25)
Absent		2(50)	3(75)	-	-
CAH	1				
Normal		0	0	1(100)	0
Rudimentary		1(100)	1(100)	0	0
Ambiguous		0	0	0	1(100)

Normal: Full length vagina, Rudimentary: Blind ended, Blind ended refers to vagina only.

**Table-III:** Treatment provided.

Treatment	No. of Cases N (%)	Diagnosis
HRT	10 (24.39)	Chromosomal aberrations
Septoplasty	2 (4.87)	Transverse vaginal septum
Hymenectomy	6 (14.63)	Imperforate hymen
Vaginoplasty	2(4.87)	Mullerian agenesis with atretic vagina
Gonadectomy	2 (4.87)	46XY gonadal dysgenesis
Phallectomy/ Clitoral reduction	2(4.87)	46XY, CAH

## DISCUSSION

We discovered that clinical features may help establish a probable diagnosis well before confirmation by sonological, hormonal or other diagnostic means. Prompt treatment is necessary not only for future fertility but also to prevent potential long term hormone related health consequences. The mean age of our patients is slightly less than reported in literature. It was 19.2 year in a study.^11^ However, our results are consistent with Bhuyan et al. who also stated mean age 17.2 years in their results.^12^ BMI of patients did not co-relate well with etiology. About three quarter of them had normal BMI and their distribution among different etiological groups was unequal.

According to various published reports previously most common cause of primary amenorrhea was chromosomal abnormalities mainly Turner syndrome^13^ but now, MRKH is ranking on top as awareness and surgical correction facilities are rising.^14^,^15^ We also recognized MRKHS as the most common. This is in contrast to a local study reported by Khyber where anatomical defects were identified as commonest and Mullerian abnormalities were only next to them.^16^ Although SSC are expected to be normal in all MRKH but only 58.3% of girls had well developed characteristics in our study (Table-I). Usually, ovaries are normal in MRKH patients as they share separate embryonic origin but we found normal ovaries in only 70.5% of participants which is in contrast with studies where only few ovarian anomalies found.^17^,^18^A total of 82.3% had rudimentary or hypoplastic uterus of varying degree while completely absent in rest of the cases which is consistent with a study by Herlin et al. who also found uterine remnants in majority.^15^

Our results revealed chromosomal aberrations as second commonest and is consistent with results of other studies.^19^,^20^ Karyotyping showed Turner/Turner mosaic in 14.6% while 9.7% of XY gonadal dysgenesis. Almost all short heighted patients of present study belonged to this group with poorly developed SSC which is in accordance to results of a study conducted in India.^21^ Those patients who didn’t have abnormality detected in history, examination and laboratory investigations have been labelled as having constitutional delay.

We diagnosed quite a good number of surgically correctable amenorrhea patients as reported by a study from Finland. 22 Corrective procedures were performed in 34% of participants. In three of our MRKH patients who were married, vaginal dilators were advised initially for atretic vagina as first line management^23^ but as results were not good so vaginoplasties performed which were associated with good patient satisfaction. Outflow problems encountered in 18.8% of participants in form of imperforate hymen and transverse vaginal septum (Table-III). Our results are almost comparable to one of local study conducted by Jabeen S et al. earlier.^24^ Presence of Y- chromosome necessitated surgical removal of gonads as risk of gonadoblastoma is about 30% after puberty in XY-females. Gonadectomies were performed in two cases after having chromosomal analysis done, confirmation of diagnosis, discussion in multidisciplinary team and patient/family counselling (Table-III). In patients with Turner syndrome estrogen replacement after explaining risks and benefits was employed to facilitate the development of secondary sexual characteristics, improve bone mineralization and keeping uterus in functional state which was a good source of psychological satisfaction in patients. They were managed in consultation with endocrinologist. Special counselling sessions were arranged for patients and their parents as efficient behavioral therapy may have resulted in spontaneous resumption of ovarian activity in hypothalamic amenorrhea than observation alone.^25^ The patients with constitutional delay and most with MRKHS were counselled and reassured about their clinical condition and given nutritional supplements. The patient with congenital adrenal hyperplasia also required multidisciplinary team management and cosmetic surgery of external genitalia.

### Limitations:

It was a single center study so data is representative of only one tertiary care from our city/ province. Other is related to lack of long term follow up. Many patients followed only for short period of time after a treatment was given and did not come afterwards to be seen for long term complications (like osteoporosis), sexual health or future fertility.

## CONCLUSION

Primary amenorrhoea is a significant problem in adolescent girls. We found MRKH syndrome as the commonest cause in our series. There is need to promptly identify the patients who need medical, surgical or psychological management. It is also required to make local strategies and guidelines for evaluation, management and long-term follow-up.

### Authors Contribution:

**SK:** Conceived and designed.

**SA:** Data interpretation and statistical analysis, Prepared the manuscript,

**FDN:** Helped manuscript writing and acquisition of data.

**RJ:** Did final review, editing.

All authors have read and approved the final manuscript and are responsible for the integrity of the study..
